# When is working memory important for arithmetic? The impact of strategy and age

**DOI:** 10.1371/journal.pone.0188693

**Published:** 2017-12-11

**Authors:** Lucy Cragg, Sophie Richardson, Paula J. Hubber, Sarah Keeble, Camilla Gilmore

**Affiliations:** 1 School of Psychology, University of Nottingham, Nottingham, United Kingdom; 2 Mathematics Education Centre, Loughborough University, Loughborough, United Kingdom; Katholieke Universiteit Leuven, BELGIUM

## Abstract

Our ability to perform arithmetic relies heavily on working memory, the manipulation and maintenance of information in mind. Previous research has found that in adults, procedural strategies, particularly counting, rely on working memory to a greater extent than retrieval strategies. During childhood there are changes in the types of strategies employed, as well as an increase in the accuracy and efficiency of strategy execution. As such it seems likely that the role of working memory in arithmetic may also change, however children and adults have never been directly compared. This study used traditional dual-task methodology, with the addition of a control load condition, to investigate the extent to which working memory requirements for different arithmetic strategies change with age between 9–11 years, 12–14 years and young adulthood. We showed that both children and adults employ working memory when solving arithmetic problems, no matter what strategy they choose. This study highlights the importance of considering working memory in understanding the difficulties that some children and adults have with mathematics, as well as the need to include working memory in theoretical models of mathematical cognition.

## Introduction

Mathematics is used extensively in our everyday lives, for example when deciding which of two items to buy, following a recipe, or splitting a dinner bill between friends. Our ability to perform mathematics relies on a number of underlying skills. These include domain-specific processes such as the knowledge and selection of appropriate strategies, but also more general factors such as language [1,2] and IQ [3]. Working memory, the manipulation and maintenance of information in mind, has been shown to play a key role in mathematics [4–6].

There are now a number of researchers, as well as commercial companies, that aim to capitalise on this relationship by training working memory in order to improve outcomes in mathematics and other academic subjects. To date it has been shown that such training can improve working memory performance as measured by standardised tests, e.g. [7–12]. However, there is very little evidence that this enhancement transfers to improved academic performance in mathematics [8,10,11,13,14]. If more is understood about the precise role that working memory plays in mathematics then it may be possible to use this to inform intervention approaches that aim to improve performance in the classroom.

Evidence for the contribution of working memory to arithmetic comes from three main sources: correlational studies demonstrating a significant relationship between working memory ability and performance on mathematical tests, e.g. [15], individual difference approaches showing poor working memory in individuals who struggle with mathematics [16–18], and experimental dual-task studies revealing an impaired ability to solve arithmetic problems when performing a concurrent working memory task [19–27]. Correlational and individual difference studies can demonstrate that working memory is important for overall mathematics achievement but cannot identify how. Experimental dual-task paradigms, however, can elucidate the specific role that working memory plays in solving arithmetic problems. This is the focus of the current study.

Dual-task paradigms work on the assumption that working memory has a limited capacity and individuals show decrements in performance when this capacity is exceeded. If task A performance is impaired by a secondary task B that loads working memory then this provides evidence that the two tasks are tapping into the same resource pool, and therefore by extension that task A involves working memory. Performance on the secondary task may also degrade. The majority of studies that have taken a dual task approach have found that it is the ability to monitor and manipulate information in working memory, often termed the ‘central executive’ [28,29] that is particularly important for arithmetic, in contrast to simply holding verbal or visuospatial information in short-term memory [19,20,24,27]. Henceforth, the use of the term working memory will refer to this higher-level executive component.

### Strategies

The extent to which arithmetic relies on working memory depends on a range of factors [5]. One of the most important of these is the strategy used to solve the arithmetic problem [22,23,25,26]. There are a number of ways in which the solution to an arithmetic problem can be reached: For example, if given the problem 5 + 7 = ?, an individual could select from several different strategies including a) directly retrieve the answer from long-term memory (*retrieval*), b) decompose the problem into a series of simpler problems, e.g. 5 + 5 = 10, 10 + 2 = 12 (*decomposition*) or c) count on seven times from 5 (*counting*). The latter two strategies are termed as procedural strategies. From a theoretical point of view, working memory is likely to be required to a greater extent for procedural as compared to retrieval strategies. Working memory is often characterised as the storage of information in the face of concurrent processing [29,30]. This typifies procedural strategies for arithmetic such as counting and decomposition where individuals are required to store interim solutions, or the number of count steps performed so far, while carrying out other procedural steps. This is not required for retrieval strategies, which only involve the single step of retrieving the solution from long-term memory.

Empirical evidence also indicates that different strategies tax working memory to different extents. Hecht [22] asked adults to verify single digit sums and then immediately report the strategy they had used. Performance on both the arithmetic problems and the secondary task was impaired under executive working memory load (random letter generation) compared to answer verification alone when participants used a counting strategy. Moreover, participants were slower to verify more difficult problems involving a larger addend. For retrieval strategies, despite overall worse performance under working memory load than the no load condition, this was not modulated by problem difficulty (measured as the proportion of participants who used a retrieval strategy). There was also no impairment on the executive working memory task when a retrieval strategy was used. Based on these results Hecht suggested that counting, but not retrieval, involves executive working memory resources. However, other single digit sum verification studies in which participants are assumed to use a retrieval strategy have demonstrated an effect of working memory load on performance [19,20,27] suggesting that working memory resources are required for retrieval strategies, but perhaps to a lesser extent.

The participants in the Hecht study [22] were able to choose the strategy they used to solve the problems. In cases such as these however strategy is often confounded with task difficulty such that a retrieval strategy would be selected for easier problems and a counting strategy for more difficult ones. As such it is not possible to separate out the influence of problem difficulty and strategy execution. To overcome this issue later studies have implemented a no-choice method in which participants are instructed to use a particular strategy [23–25,31,32]. Using this approach a central executive working memory load has been found to impair addition and subtraction for counting, decomposition and retrieval strategies but to differing extents. Some researchers have found working memory to have a greater impact on both types of procedural strategies (counting and decomposition) compared to retrieval [25] whereas others have found that a concurrent working memory load impairs counting to a greater extent than both retrieval and decomposition [23]. It may be that decomposition relies less on working memory in adults because they are able to employ retrieval strategies to complete some steps of the procedure thereby reducing the cognitive load.

### Developmental changes

Given that there is a shift from the use of procedural strategies to retrieval strategies during childhood [33,34], as well as an increase in the accuracy and efficiency of arithmetic strategy use with age [35], it seems likely that the role of working memory in arithmetic may also change during development. Indeed, the processing demands of arithmetic have been found to differ depending on the age of the participant. Kaye, deWinstanley, Chen and Bonnefil [36] asked 7-, 9-, 11-year-olds and adults to verify single digit addition problems and concurrently respond to auditory probes presented at different times during the processing of the arithmetic problem. They found a reduction in the impact of the probe detection task with age suggesting greater efficiency in arithmetic processing. This study did not directly test whether these processing demands involved working memory. However, it has been shown that the simple storage of verbal or visuospatial information in memory does impair arithmetic performance in children of this age range [37,38].

To our knowledge, only two experimental studies to date have explored the extent to which children rely on executive working memory to solve arithmetic problems. Thomas, Zoelch, Seitz-Stein, and Schumann-Hengsteler [39] asked 9- and 10-year-olds to complete addition problems that either did or did not involve crossing a decade boundary while completing a random number generation or neutral tapping task (Experiment 2). They found that the addition problems were completed more slowly when combined with the number generation task than the neutral tapping task, particularly when the problem involved a carry-over. This study provides evidence that executive working memory is required when children perform basic arithmetic. However, it does not inform us as to how children engage working memory when using different strategies to solve addition problems.

We would expect children to rely on working memory to a greater extent than adults when doing arithmetic. Children are less efficient at solving arithmetic problems than adults and therefore the necessary information to complete the task has to be stored in mind for longer, making it more susceptible to interference and forgetting. The recruitment of working memory across different strategies may also differ for children as compared to adults. Whereas both adults and children are likely to require more working memory resources for counting as compared to retrieval, the pattern for decomposition may differ. Adults are able to make adaptive strategy choices and thereby select a decomposition strategy that minimizes working memory demands. Children, on the other hand, are likely to use less sophisticated and efficient decomposition strategies with a heavier working memory load. For example, when faced with the problem 8 + 9, adults may be more likely to decompose this into 8 + 10–1, whereas children may take the more resource-demanding route of 8 + 2 + 7.

Initial evidence suggests that children do indeed show a different pattern of reliance on working memory across strategies compared to adults. Imbo & Vandierendonck [26] gave 10-, 11-, and 12-year-olds a series of single digit addition problems and asked them to solve them either by counting, retrieval or decomposition. The sums were solved either under an executive working memory load (choice RT task) or no load conditions. In contrast to previous findings with adults, for whom working memory load appears to have greatest impact on counting, the effect of load was larger on children’s decomposition RTs than their counting and retrieval speed, which showed a similar effect of load. These results suggest that whereas adults rely most on working memory when counting, children find decomposition strategies the most resource demanding. However, this study did not directly compare children and adults in order to determine if this is indeed the case.

Moreover, it seems odd that the effect of working memory load was equivalent for retrieval and counting in this group of children when the cognitive demands of counting appear to be so much higher. This finding may be due to the lack of a control condition in this task, and as a result what is actually being measured is the demands of performing two tasks concurrently rather than being specific to the competing demands on working memory. Performing any two tasks concurrently is known to lead to a decrement in performance [40,41]. In many domains dual task studies include a control task with a similar format to the main secondary task but excluding the main process of interest. For example, foot tapping is often used as a control for articulatory suppression, e.g. [38]. The inclusion of a control condition allows the impact of working memory load to be more precisely assessed. If task A performance is impaired by secondary task B that loads working memory, over and above that of secondary task C with no working memory load, then this provides more convincing evidence that task A involves working memory and that the impairment isn’t simply due to the demands of performing two tasks concurrently. The use of a control task in dual task arithmetic studies is rare and therefore in Imbo and Vandierendonck’s study, as in many others, it is not possible to determine if it is the working memory component of the secondary task that is impairing arithmetic performance over and above the general task demands. The current study addressed these issues by including a control task and directly comparing children and adults in order to ascertain if the extent to which different arithmetic strategies require working memory resources really does change with age.

### Working memory domain

Within the developmental psychology literature there has been growing debate as to whether the ability to monitor and manipulate verbal or visuospatial information in mind is of greater relevance for successful arithmetic achievement. While some studies, including a large meta-analysis, have demonstrated a stronger link between verbal working memory and mathematical achievement [15,42], others suggest that visuospatial working memory is of greater importance [43–47]. This latter set of findings is consistent with a large body of evidence linking general visuospatial skills to mathematics achievement [48]. Many studies to date have not included both verbal and visuospatial versions of short-term and working memory tasks, making it difficult to determine their relevant contribution. A recent study that used parallel verbal and visuospatial tasks in participants aged between 8 and 25 years found that the contribution of verbal and visuospatial working memory was in fact very similar [49].

The majority of dual-task studies that have investigated the role of working memory in performing arithmetic have used central executive tasks within the verbal domain, typically using a random letter or number generation task [22,50]. These tasks interfere with solving arithmetic problems, which has been taken as evidence that domain-general central executive resources are required, however it is possible that only the monitoring and manipulation of verbal information is required for arithmetic.

There is much less evidence regarding the contribution of visuospatial working memory. Hubber, Gilmore and Cragg [23] used a visuospatial working memory n-back task (Experiment 1) and found that a concurrent visuospatial working memory load interfered with arithmetic performance, particularly for counting. However, a follow-up experiment which compared interference from a visuospatial short-term memory task (remembering the location of dots within a grid) and a central executive random letter generation task suggested that it was the central executive demands of the n-back task that interfered with arithmetic performance, rather than the visuospatial nature of the task. This indicates that the working memory requirements of solving arithmetic problems may indeed be domain-general rather than domain-specific.

To date, no dual-task study has used both verbal and visusopatial secondary tasks that both include a central executive load. Based on correlational findings, which show different relationships with mathematics achievement for verbal and visuospatial central executive tasks, it is plausible that differential relationships may also be found within an experimental paradigm. Moreover, it may be that the type of working memory that is relied upon differs depending on the strategy used. For example, counting may rely more heavily on verbal than visuospatial working memory resources due to its heavy reliance on verbal codes. Similarly, numerical facts are thought to be stored in a verbal code [51] and therefore directly retrieving facts may also rely on verbal working memory to a greater extent. In contrast, decomposition strategies may require more visuospatial working memory resources in order to visualise number lines and break sums down into smaller parts.

### The current study

This study investigated the extent to which working memory requirements for different arithmetic strategies differ with age and working memory domain. Participants were asked to solve a series of addition problems by either counting, breaking the sum down into smaller parts (decomposition) or retrieving the answer from memory (retrieval). Three age groups were studied: 9-11-year-olds, who are still learning number facts and receiving instruction in decomposition strategies. 12-14-year-olds, who are expected to be familiar with all three strategies but may still be improving in their application, and young adults, who are expected to be proficient in the use of all three strategies.

In order to separate working memory interference from the general interfering demands of completing a secondary task, three levels of dual task load were included. In the no load condition participants completed the arithmetic problems with no secondary task. In the control load condition participants monitored a sequence of verbally or visually presented information and had to respond to a particular stimulus. In the working memory load condition the participants monitored the same sequence of information but had to respond to a particular pattern in the sequence. This required the participant to continuously update the final few items of the sequence in memory. The participants completed both verbal and visuospatial versions of the control and working memory tasks.

It was predicted that performance on the arithmetic problems would be slower and less accurate in the control load condition than the no load condition, due to the demands of performing two tasks at once. More importantly, performance on the arithmetic problems was expected to be slower and less accurate in the working memory load condition than the control load condition, due to competing demands for working memory resources. The main question of interest in this dual task study was how working memory load interacted with strategy, and whether this changed with age and working memory domain. For adults, we predicted that counting strategies would show the largest deficit under working memory load, followed by decomposition and then retrieval strategies. We predicted that the children would show a greater effect of working memory load than the adults, particularly for procedural strategies. We hypothesised that the 9-11-year-olds would show a greater deficit under working memory load for both counting and decomposition strategies as compared to retrieval. It was not clear if the 12-14-year-olds would bear greater resemblance to the 9-11-year-olds or to the adults. With regards to working memory domain, we predicted an interaction between strategy, working memory load and working memory domain, such that a verbal working memory load would interfere with counting and retrieval strategies to a greater extent than a visuospatial working memory load, which would interfere more with a decomposition strategy.

## Method

### Participants

138 participants took part in this experiment. Seven 9-11-year-olds, six 12-14-year-olds and three adults were excluded for failing to complete the task or not sticking to the required strategy. This left a total of 44 9-11-year-olds (M = 10.1 years, SD = 2.37; 19 male), 39 12-14-year-olds (M = 13.1 years, SD = 0.90; 19 male) and 39 adults (M = 19.6 years, SD = 0.90; 13 male). The children attended primary (9-11-year-olds) and secondary (12-14-year-olds) schools in predominantly white British, low to average socio-economic status neighbourhoods of UK cities. The adults were undergraduate psychology students. All adults provided written informed consent and received course credit for participation. Informed written parental consent was received for all child participants, who gave verbal assent. Children received a certificate for taking part. The study was approved by the University of Nottingham School of Psychology ethics committee.

### Equipment and materials

The experimental tasks were created and controlled using E-Prime software and are available to download from osf.io/fzbka. The tasks were run on a Samsung laptop computer. Responses to the addition problems were made using an external numeric keypad whilst responses to the secondary working memory tasks were made using the laptop’s in-built mouse.

### Addition task

Participants were required to answer a series of two-addend addition problems presented horizontally in the centre of the screen. Nine lists of 20 problems were created with the same mean sum total. Within each problem set, half of the problems comprised two single digit operands between 2 and 9 and half comprised a double-digit operand between 13 and 29 (20 omitted) on the left and single-digit operand between 3 and 9 on the right. Tie problems were omitted. Within each of these problem types half of the problems crossed a decade boundary and half did not. A full list of all problems can be found in S1 Appendix.

The 12-14-year-olds and adults entered the answers themselves using the numerical keypad. The 9-11-year-olds said the answer out loud and the experimenter entered the answers on the keypad. The experimenters were given full training to ensure that this was an accurate method of recording response times. Pilot testing of the study protocol in 29 adults and 15 children was used to verify the reliability of this method and ensure that the results were comparable across age groups.

### Visuospatial secondary task

This task, also described in Hubber et al. [23], consisted of two rows of four horizontal boxes, with one row above and one row below the arithmetic problems. Different boxes turned red, randomly and one at a time, for 2 seconds and participants had to respond, using the mouse, when a specified pattern was noted. Three dual task conditions were used: no load, where the boxes were present on screen but none turned red and participants only had to answer the sums; a control load, where participants had to click the mouse when the box second from left on the top row turned red; and a working memory load, where participants had to respond when the box that turned red was the same as the box two items previously in the sequence. For both the control and working memory loads an event requiring a response occurred at least on every sixth box turning red. If participants missed a response, an auditory ‘beep’ was heard, to remind them to pay attention to the secondary task.

### Verbal secondary task

A string of animal names were presented aurally through headphones in a random order at a rate of one word every 3 seconds. Participants had to respond, using the mouse, when a specified pattern was noted. Three dual task conditions were used: no load, where no animal names were heard and participants only had to answer the sums; a control load where participants had to respond when they heard the word ‘dog’; and a working memory load where participants had to respond when the animal name was the same as the one presented previously. For both the control and working memory loads, an event requiring a response occurred at least on every sixth word. If participants missed a response, the boxes surrounding the sums flashed red for 100 milliseconds, to remind them to pay attention to the secondary task.

### Procedure

The participants were tested in a quiet room either at their school or at the university. The verbal and visuospatial versions of the task were split across two sessions for the 9-11-year-olds, with a maximum of three days between sessions. Adults and 12-14-year-olds completed both versions in one session. The order in which the verbal and visuospatial versions were completed was counterbalanced across participants. The procedure was similar to that described in Experiment 1 of Hubber et al. [23]. The within-participants design required participants to answer 20 addition problems in each possible combination of strategy and dual task load. This resulted in a total of nine combinations (retrieval with no load, control load, working memory load; decomposition with no load, control load, working memory load; counting with no load, control load, working memory load) and therefore 180 problems for each of the verbal and visuospatial versions.

For both versions participants began with twenty practice problems which could be answered using any strategy. They then practised the control and working memory tasks, which could be repeated if necessary, before moving on to the test trials. At the start of each strategy block (counting, decomposition, retrieval), participants completed eight practice problems before moving on to the three dual task load sub-blocks. The order of strategy blocks was assigned randomly, whilst the order of dual task load sub-blocks (no load, control load, working memory load) was counterbalanced across participants. The combination of each problem set with the strategy and dual task load conditions was counterbalanced across participants.

On each trial the arithmetic problems were presented in the centre of the screen between the two rows of boxes used for the visuospatial task. The arithmetic problems remained on screen whilst participants used the required strategy to work out the answer. Reaction time was measured from the time the problem appeared until the enter key was pressed to indicate the problem had been solved. After the answer had been keyed in the enter key was pressed again, which triggered the appearance of the next problem. The continuous control and working memory tasks started when the first problem of each block was presented on the screen and ended when the enter key was pressed to respond to the final problem of the block. No new stimuli were presented while the responses to the arithmetic problems were entered but participants were required to remember the previous stimulus across this short delay. Participants were told to give equal attention to the addition problems and the secondary task. At the end of each set of 20 problems, participants were asked, on a scale of 1 to 5, how many of the problems they had used the required strategy for, where 1 was ‘hardly any’ and 5 was ‘almost all’. The experimenter then entered their response using the numeric keypad. Any participants who reported that they used the strategy for ‘hardly any’ problems were excluded from data analysis (one adult and one 12-14-year-old).

## Results

### Arithmetic problems

Mean accuracy and log mean RT for each strategy and dual task load combination per participant were calculated for performance on the arithmetic problems. Log transformed means were used in order to account for baseline differences in speed between age groups, e.g. [52,53]. Back-transformed means are presented for ease of interpretability. Separate four-way mixed measures ANOVAs were performed for accuracy and RT data. The between-subjects factor was age group (9–11 years, 12–14 years, adults) and the within-subject factors were working memory domain (verbal, visuospatial), strategy (counting, decomposition, retrieval), and dual task load (no load, control load, working memory load). Descriptive statistics are provided in Table 1. Degrees of freedom were corrected using Greenhouse-Geisser estimates of sphericity where necessary. Significant interactions were followed up with tests of simple main effects and Bonferroni corrected post-hoc t-tests as appropriate.

**Table 1:** Mean accuracy (%) and back-transformed mean RT data (ms) with 95% confidence intervals (CI) for the arithmetic problems by domain, age group, strategy and dual task load.

Working memory domain	Age group	Strategy	Dual task load	Mean accuracy (95% CI)	Back-transformed mean RT (95% CI)
Verbal	9-11-year-olds	Counting	No load	0.95 (0.94–0.97)	3784 (3436–4169)
			Control load	0.92 (0.89–0.94)	4375 (3926–4864)
			Working memory load	0.91 (0.88–0.93)	4667 (4217–5176)
		Decomposition	No load	0.92 (0.89–0.94)	3184 (2773–3648)
			Control load	0.87 (0.84–0.90)	3690 (3266–4178)
			Working memory load	0.84 (0.81–0.88)	3890 (3428–4416)
		Retrieval	No load	0.86 (0.82–0.89)	2761 (2404–3162)
			Control load	0.83 (0.78–0.87)	3221 (2838–3656)
			Working memory load	0.78 (0.74–0.82)	3396 (2972–3873)
	12-14-year-olds	Counting	No load	0.94 (0.93–0.96)	3467 (3133–3846)
			Control load	0.91 (0.89–0.94)	3573 (3192–4009)
			Working memory load	0.90 (0.87–0.93)	4064 (3648–4539)
		Decomposition	No load	0.92 (0.90–0.94)	1663 (1439–1923)
			Control load	0.88 (0.85–0.91)	2118 (1862–2415)
			Working memory load	0.84 (0.80–0.87)	2371 (2070–2710)
		Retrieval	No load	0.82 (0.78–0.86)	1156 (1000–1337)
			Control load	0.78 (0.73–0.83)	1510 (1318–1726)
			Working memory load	0.76 (0.71–0.80)	1614 (1403–1862)
	Adults	Counting	No load	0.97 (0.95–0.99)	2377 (2143–2630)
			Control load	0.95 (0.92–0.97)	2612 (2333–2931)
			Working memory load	0.92 (0.89–0.94)	2812 (2523–3133)
		Decomposition	No load	0.95 (0.93–0.97)	1483 (1282–1714)
			Control load	0.93 (0.90–0.96)	1675 (1469–1910)
			Working memory load	0.91 (0.87–0.94)	1858 (1622–2128)
		Retrieval	No load	0.90 (0.86–0.94)	830 (718–959)
			Control load	0.86 (0.81–0.91)	1021 (891–1167)
			Working memory load	0.84 (0.79–0.88)	1107 (962–1276)
Visuospatial	9-11-year-olds	Counting	No load	0.94 (0.91–0.96)	3855 (3499–4236)
			Control load	0.92 (0.89–0.94)	4159 (3767–4592)
			Working memory load	0.90 (0.87–0.92)	4977 (4416–5610)
		Decomposition	No load	0.94 (0.92–0.97)	3184 (2805–3622)
			Control load	0.90 (0.87–0.93)	3606 (3177–4102)
			Working memory load	0.87 (0.84–0.90)	4236 (3707–4842)
		Retrieval	No load	0.86 (0.81–0.90)	2799 (2466–3177)
			Control load	0.82 (0.77–0.86)	3090 (2729–3499)
			Working memory load	0.77 (0.72–0.81)	3524 (3112–3999)
	12-14-year-olds	Counting	No load	0.94 (0.92–0.97)	2897 (2618–3206)
			Control load	0.91 (0.89–0.94)	3184 (2864–3540)
			Working memory load	0.86 (0.83–0.89)	3690 (3251–4198)
		Decomposition	No load	0.91 (0.88–0.93)	1589 (1387–1820)
			Control load	0.89 (0.86–0.92)	1959 (1710–2244)
			Working memory load	0.83 (0.79–0.86)	2399 (2080–2767)
		Retrieval	No load	0.78 (0.73–0.82)	1194 (1045–1368)
			Control load	0.79 (0.74–0.83)	1406 (1230–1603)
			Working memory load	0.72 (0.67–0.77)	1671 (1466–1910)
	Adults	Counting	No load	0.95 (0.93–0.98)	2339 (2113–2588)
			Control load	0.94 (0.91–0.96)	2449 (2203–2723)
			Working memory load	0.91 (0.88–0.94)	2992 (2630–3396)
		Decomposition	No load	0.95 (0.92–0.97)	1449 (1265–1656)
			Control load	0.92 (0.89–0.95)	1675 (1462–1919)
			Working memory load	0.87 (0.84–0.91)	2042 (1770–2355)
		Retrieval	No load	0.90 (0.85–0.94)	815 (713–933)
			Control load	0.86 (0.81–0.90)	1035 (908–1183)
			Working memory load	0.84 (0.79–0.89)	1303 (1143–1489)

#### Accuracy

Significant main effects of age group, *F* (2, 119) = 5.98, *p* = .003, η_p_^2^ = 0.91, and strategy, *F* (1.32, 157) = 98.0, *p* < .001, η_p_^2^ = 0.45 were qualified by a significant age group x strategy interaction, *F* (2.64, 157) = 3.07, *p* = .035, η_p_^2^ = 0.05. All participants were less accurate when retrieving answers than when using a decomposition strategy (all p < .004) but only the 9-11-year-olds and 12-14-year-olds were less accurate at using decomposition than counting strategies (all p < .001). There was a main effect of dual task load, *F* (2, 238) = 123, *p* < .001, η_p_^2^ = 0.51, as arithmetic performance was less accurate when also completing the control load task (p < .001; M = .880, 95% CI [.867, .894]) than when completing the problem alone (M = .910, 95% CI [.898, .922]). Performance was significantly more impaired when concurrently performing the working memory load task (M = .846, 95% CI [.832, .861]) as compared to the control load task (p < .001). The two-way interaction between strategy and dual task load and the three-way interaction with age group did not reach significance for accuracy, indicating that the effect of load on accuracy was similar across age groups and strategies. No other main effects or interactions reached significance. As pointed out by one of the reviewers, the influence of working memory on strategies could differ depending on the size of the operands in the arithmetic problem. To investigate this, we repeated the analyses including problem size (single digit operands, one double digit operand) as a within-subject factor. There was a significant three-way interaction between strategy, working memory load and problem size, *F* (3.56, 423) = 3.05, *p* = .021, η_p_^2^ = 0.03. Post-hoc comparisons showed that for the procedural strategies (counting and decomposition) there was no significant difference between the control load and working memory load conditions for the single digit problems (counting: control; M = .949, 95% CI [.938, .959], working memory; M = .934, 95% CI [.922, .947], *p =* .*15*. Decomposition: control; M = .917, 95% CI [.902, .932], working memory; M = .906, 95% CI [.888, .932], *p* = .55), but there was for the double digit problems (counting: control; M = .898, 95% CI [.881, .914], working memory; M = .861, 95% CI [.843, .879], *p* < .001. Decomposition: control; M = .879, 95% CI [.858, .899], working memory; M = .813, 95% CI [.791, .835], p < .001). For retrieval there was a significant difference between the control load and working memory load conditions for both single (control; M = .867, 95% CI [.844, .890], working memory; M = .834, 95% CI [.811, .8858]) and double digit (control; M = .773, 95% CI [.743, .803], working memory; M = .730, 95% CI [.702, .759]) problems (both p < .01). This is consistent with the prediction that the role of WM on procedural strategies is larger for more difficult problems. There was no interaction with age group however, *F* (7.11, 395) = 1.13, *p* = .34, suggesting that this was the case for all age groups and not most apparent in the younger children.

#### RT

**Fig 1:**
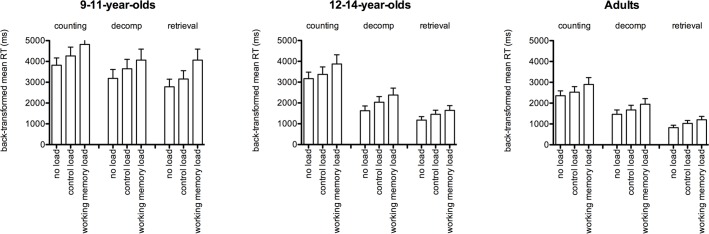
Three-way interaction between strategy, dual-task load and age group. Error bars represent 95% confidence intervals.

Significant main effects of age group, *F* (2, 119) = 76.0, *p* < .001, η_p_^2^ = 0.56, strategy, *F* (1.53, 182) = 235, *p* < .001, η_p_^2^ = 0.66, and dual task load, *F* (1.90, 226) = 485, *p* < .001, η_p_^2^ = 0.80, as well as significant two-way interactions between strategy and age group, *F* (3.05, 182) = 20.3, *p* < .001, η_p_^2^ = 0.25, dual task load and age group, *F* (3.79, 226) = 3.59, *p* = .008, η_p_^2^ = 0.06, and strategy and dual task load, *F* (3.67, 437) = 10.5, *p* < .001, η_p_^2^ = 0.08, were qualified by a significant three-way interaction between strategy, dual task load and age group, *F* (7.35, 437) = 3.75, *p* < .001, η_p_^2^ = 0.06 (Fig 1). Tests of simple main effects demonstrated significant effects of load for all strategies and age groups (all p < .001, η_p_^2^ > .32), with slower performance on the control load condition compared to the no load condition, and even slower performance on the working memory load condition. Follow-up t-tests suggested that the interaction arose due to smaller effects of the control load on counting in the adults (difference = 174 ms, p = .01) and 12-14-year-olds (difference = 203 ms, p = .029) than in the 9-11-year-olds (difference = 446 ms, p < .001). For RT, including problem size as a factor did not moderate the main contrasts of interest: differences between the control load and working memory load conditions were significant across all problem sizes and strategies (all p < .001).

There was no main effect of working memory domain on RT, *F* (1, 119) < 1, *p* = .67, however there was a significant domain x dual task interaction, *F* (1.88, 224) = 18.7, *p* < .001, η_p_^2^ = 0.14. Tests of simple main effects demonstrated that while there was no effect of domain in the no load condition, *F* (1, 119) = 1.99, *p* = .16, participants were slower under conditions of verbal load (M = 2399, 95% CI [2265, 2547]) as compared to visuospatial load (M = 2291, 95% CI [2168, 2421]) in the control condition, *F* (1, 119) = 6.58, *p* = .012, η_p_^2^ = 0.05, but slower under visuospatial load (M = 2742, 95% CI [2582, 2917]) as compared to verbal load (M = 2606, 95% CI [2460, 2761]) in the working memory condition, *F* (1, 119) = 5.67, *p* = .019, η_p_^2^ = 0.05. There was also a significant domain x strategy interaction, *F* (2, 238) = 3.57, *p* = .030, η_p_^2^ = 0.03. Tests of simple main effects indicated slightly slower performance under conditions of verbal load (M = 3443, 95% CI [3251, 3648]) compared to visuospatial load (M = 3304, 95% CI [3112, 3499]) when participants used a counting strategy, *F* (1, 119) = 3.92, *p* = .050, η_p_^2^ = 0.03, but not when they used a decomposition (verbal = 2296, 95% CI [2128, 2472]; visuospatial = 2301, 95% CI [2138, 2477], *F* (1, 119) < 1, *p* = .91) or retrieval strategy (verbal = 1626, 95% CI [1507, 1758]; visuospatial = 1656, 95% CI [1542, 1782], *F* (1, 119) < 1, *p* = .38). The three-way interaction between domain, strategy and dual task load did not reach significance, *F* (4, 476) < 1, *p* = .87.

### Secondary tasks

In order to analyse performance on the secondary control and working memory tasks, d’ was calculated based on the accuracy of responding to targets. This was done separately for each strategy in each working memory domain. Data were not recorded for 5 adults and five 12-14-year-olds on these measures leaving a sample of forty-four 9-11-year-olds, thirty-four 12-14-year-olds and 34 adults. A mixed measures ANOVA was performed with working memory domain (verbal, visuospatial), task (control, working memory) and strategy (counting, decomposition, retrieval) as within-subject factors and age group (9-11-year-olds, 12-14-year-olds, adults) as a between-subjects factor. Descriptive statistics are provided in Table 2. Degrees of freedom were corrected using Greenhouse-Geisser estimates of sphericity where necessary. Significant interactions were followed up with tests of simple main effects and Bonferroni corrected post-hoc t-tests as appropriate.

**Table 2:** Mean accuracy (%) and back-transformed mean RT data (ms) with 95% confidence intervals (CI) for the secondary task by domain, dual task load, age group and strategy.

Working memory domain	Dual task load	Age group	Strategy	d’ (95% CI)
Verbal	Control load	9-11-year-olds	Counting	2.51 (2.33–2.70)
			Decomposition	2.50 (2.30–2.70)
			Retrieval	2.07 (1.85–2.30)
		12-14-year-olds	Counting	2.86 (2.65–3.08)
			Decomposition	2.46 (2.23–2.69)
			Retrieval	2.20 (1.94–2.45)
		Adults	Counting	2.62 (2.41–2.84)
			Decomposition	2.36 (2.13–2.59)
			Retrieval	1.89 (1.63–2.14)
	Working memory load	9-11-year-olds	Counting	2.36 (2.15–2.56)
			Decomposition	2.09 (1.87–2.31)
			Retrieval	1.80 (1.57–2.03)
		12-14-year-olds	Counting	2.52 (2.28–2.75)
			Decomposition	2.14 (1.89–2.39)
			Retrieval	1.62 (1.36–1.88)
		Adults	Counting	2.44 (2.21–2.68)
			Decomposition	2.19 (1.93–2.44)
			Retrieval	1.59 (1.33–1.86)
Visuospatial	Control load	9-11-year-olds	Counting	2.65 (2.44–2.87)
			Decomposition	2.56 (2.36–2.77)
			Retrieval	2.42 (2.21–2.64)
		12-14-year-olds	Counting	2.51 (2.26–2.75)
			Decomposition	2.45 (2.22–2.68)
			Retrieval	2.08 (1.83–2.33)
		Adults	Counting	2.84 (2.59–3.09)
			Decomposition	2.66 (2.43–2.89)
			Retrieval	2.34 (2.10–2.59)
	Working memory load	9-11-year-olds	Counting	1.12 (0.90–1.34)
			Decomposition	0.88 (0.66–1.11)
			Retrieval	0.86 (0.68–1.03)
		12-14-year-olds	Counting	1.55 (1.29–1.80)
			Decomposition	1.35 (1.09–1.60)
			Retrieval	1.08 (0.88–1.28)
		Adults	Counting	1.87 (1.62–2.13)
			Decomposition	1.74 (1.48–2.00)
			Retrieval	1.57 (1.38–1.77)

Significant main effects of domain, *F* (1, 109) = 58.2, *p* < .001, η_p_^2^ = 0.35, and working memory load, *F* (1, 109) = 454, *p* < .001, η_p_^2^ = 0.81, and significant two-way interactions between domain and working memory load, *F* (1, 109) = 137, *p* < .001, η_p_^2^ = 0.56, domain and age group, *F* (1, 109) = 13.2, *p* < .001, η_p_^2^ = 0.20, and age group and working memory load, *F* (1, 109) = 10.9, *p* < .001, η_p_^2^ = 0.17. were qualified by a significant three-way interaction between domain, age group and working memory load, *F* (2, 109) = 10.2, *p* < .001, η_p_^2^ = 0.16. Tests of simple main effects showed that, for the control load, performance was significantly worse on the verbal task compared to the visuospatial task in 9-11-year-olds, *F* (1, 109) = 4.62, *p* = .034, η_p_^2^ = 0.04 and adults, *F* (1, 109) = 11.0, *p* = .001, η_p_^2^ = 0.09, whereas there was no significant difference in 12-14-year-olds, *F* (1, 109) = 2.70, *p* = .104, η_p_^2^ = 0.02. For working memory load, performance was significantly worse on the visuospatial task compared to the verbal task in all age groups (9-11-year-olds: *F* (1, 109) = 161, *p* < .001, η_p_^2^ = 0.60; 12-14-year-olds: *F* (1, 109) = 57.2, *p* < .001, η_p_^2^ = 0.34; adults: *F* (1, 109) = 11.6, *p* = .001, η_p_^2^ = 0.10.

A significant main effect of strategy was qualified by a small but significant strategy by domain interaction, *F* (2.0, 217.5) = 3.02, *p* < .001, η_p_^2^ = 0.07. Follow-up t-tests indicated that performance on the visuospatial task was worse than on the verbal task for all strategies (all p < .035). For the verbal task, performance was best when using a counting strategy, followed by a decomposition strategy, then a retrieval strategy, with significant differences between all three strategies (all *p* < .001). For the visuospatial task there was no significant difference between visuospatial task performance when using a counting or decomposition strategy, but performance was significantly worse when using a retrieval strategy compared to both counting (*p* < .001) and decomposition (*p* = .002).

## Discussion

This study used a dual-task design to investigate the extent to which working memory requirements for different arithmetic strategies change with age and working memory domain. A control task was included in order to isolate the effects of central executive working memory load from the general dual-task demands of performing two tasks at once. The results suggested that the central executive component of working memory is important for solving arithmetic problems by counting, decomposition and retrieval strategies in individuals between 9 and 25 years of age.

### Strategies

Previous research has indicated that the extent to which arithmetic relies on working memory depends on the strategy used to solve the problem. We compared the effect of working memory load on both the speed and accuracy of problem solutions when participants solved them by a) directly retrieving the answer from memory, b) by decomposing the problem into a series of smaller problems, or c) by counting. Following prior studies [22,23,25,26] we expected that procedural strategies (counting and decomposition) would rely on working memory to a greater extent than retrieval.

The results for both accuracy, which is not always considered in dual-task studies, and RT showed that working memory interfered with all three strategies to a similar extent, as evidenced by slower and less accurate performance in the working memory load condition compared to the control load condition. Including problem size in the model suggested that, for accuracy at least, the effect of working memory load on procedural, but not retrieval, strategies is influenced by problem size. However, it did not alter the fact that working memory load resulted in a decrement in performance in all three strategies.

This finding contrasts with previous studies which found a greater effect of load for procedural strategies compared to retrieval strategies [22,23,25,26]. There is no clear explanation for the difference in findings across these studies. The present study used the same arithmetic problems and drew adult participants from the same population as Hubber et al., [23]. It does however show that the differential effects of load on different arithmetic strategies may not be as clear as previously thought. One possibility is that there was variation in the counting and decomposition methods used by participants, e.g. counting all vs. counting on, which may have been employed to different extents under different dual task loads, resulting in the effects of working memory load on procedural strategies being underestimated. Further studies coding the exact way different strategies were executed are required in order to test this hypothesis.

Some researchers have suggested that retrieval does not involve working memory resources [22]. In contrast, our findings imply that the central executive component of working memory plays an important role in the general attentional requirements of retrieving facts from memory. This is consistent with working memory models such as the time-based resource-sharing account (TBRS, [54]) which views working memory as a general attentional resource that is needed to activate knowledge in long-term memory. Indeed, Barrouillet & Lépine [55] found that 8-9-year-olds with high working memory capacity were more likely, and also faster, to solve single-digit addition problems using retrieval than children with low working memory capacity. This suggests that, for children at least, working memory ability is utilised in arithmetic even when very simple problems are retrieved from long-term memory.

### Developmental changes

One of the main aims of this study was to determine if the role of working memory in arithmetic changes during the course of development. We expected that children would be more affected by the dual-task working memory load than adults as they would be less efficient at solving the arithmetic problems. Moreover, we predicted that, for adults, counting would show a greater deficit under working memory load than retrieval, with decomposition falling somewhere between the two. For the 9-11-year-olds we predicted a different pattern to the adults. It was hypothesised that counting would show a greater deficit under working memory load than retrieval, but that decomposition would also show a large deficit under working memory load as children are less able to make adaptive strategy choices to minimise cognitive resources. It was not clear if the 12-14-year-olds would bear greater resemblance to the 9-11-year-olds or the adults.

Our results did not support these predictions. For accuracy there was no interaction between strategy, dual task load and age, indicating that the effect of working memory load on the accuracy of arithmetic performance was similar for all age groups across all three strategies. A three-way interaction between strategy, dual task load and age was evident for RTs, however this was driven by differences in the impact of the control load on arithmetic performance, rather than the contribution of working memory load. Thus, as for accuracy, it appeared that working memory had a similar effect on the speed of arithmetic performance in all age groups and across all strategies.

Our predictions regarding developmental changes were largely based on theoretical assumptions as there is currently very little empirical evidence from experimental studies comparing the role of working memory in arithmetic in children and adults. Although these results do not support our predictions they are consistent with other correlational findings that indicate that the relationship between working memory and arithmetic is stable from older childhood into adulthood [49,56]. Taken together, these empirical findings suggest that the processes involved in the online performance and solution of arithmetic operations are similar no matter what the age of the participant. This suggests that theories of the role of working memory in performing or ‘doing’ arithmetic can be applied from mid-childhood through to young adulthood. However, this does not mean that the processes involved in learning new mathematical material may not be influenced by the age of the participant.

### Working memory domain

To date there has been very little experimental research examining the contribution of verbal and visuospatial working memory in the performance of arithmetic. We studied the impact of a verbal or visuospatial working memory load while participants solved arithmetic problems using one of three different strategies and measured the impact of the working memory load on arithmetic performance as well as performance on the secondary tasks themselves. Unfortunately, the analysis of the secondary task performance indicated that 9-11-year-olds and adults performed worse on the verbal control task than the visuospatial control task, and that all participants performed worse on the visuospatial working memory task than the verbal working memory task. This suggests that, despite our efforts, the verbal and visuospatial secondary tasks were not matched in difficulty, and suggests that the central executive demands were greater in the visuospatial working memory task than the verbal working memory task [57]. As a result of this it is impossible to draw accurate conclusions on the relative impact of verbal and visuospatial working memory on arithmetic performance. Further research with carefully matched working memory measures is needed in order to test whether verbal and visuospatial working memory differentially affect counting, decomposition and retrieval arithmetic strategies.

## Conclusions

The findings from this study clearly demonstrate that both children and adults rely heavily on working memory even for simple arithmetic, no matter which strategy they choose. This highlights the need to consider the important role of working memory both in theoretical models of mathematical cognition and in understanding the difficulties that some children and adults have with mathematics. Many theories of mathematical cognition do not integrate their models into a broader system of domain-general cognitive processes and skills. Moreover, when individuals are found to struggle with mathematics it is their domain-specific problems, such as poor representations of number or use of less sophisticated strategies, which are typically focussed upon. Moving forward it is essential to address the interactions that are likely to exist between domain-specific and domain-general systems. For example, individuals may be able to compensate for poor mathematical knowledge with good working memory capacity [58], and executive function systems may mediate the relationship between basic numerical representations and mathematics outcomes [59]. Further integration across research into both the domain-specific and domain-general cognitive systems involved in mathematics performance is critical in order to understand this complex skill and the reasons why many individuals struggle with it.

## Supporting information

S1 Appendix List of addition problems for all conditions. (PDF)

## References

[1] Donlan C, Cowan R, Newton E, Lloyd D. The role of language in mathematical development: Evidence from children with specific language impairments☆. Cognition. 2007;103(1):23–33. doi: 10.1016/j.cognition.2006.02.007 16581052 10.1016/j.cognition.2006.02.007

[2] LeFevre J-A, Fast L, Skwarchuk S-L, Smith-Chant BL, Bisanz J, Kamawar D, et al Pathways to Mathematics: Longitudinal Predictors of Performance. Child Dev. 2010 11;81(6):1753–67. doi: 10.1111/j.1467-8624.2010.01508.x 21077862 10.1111/j.1467-8624.2010.01508.x

[3] Mayes SD, Calhoun SL, Bixler EO, Zimmerman DN. IQ and neuropsychological predictors of academic achievement. Learn Individ Differ. 2009;19(2):238–41.

[4] Cragg L, Gilmore C. Skills underlying mathematics: The role of executive function in the development of mathematics proficiency. Trends Neurosci Educ. 2014 6;3(2):63–8.

[5] DeStefano D, LeFevre J. The role of working memory in mental arithmetic. Eur J Cogn Psychol. 2004;16(3):353–86.

[6] Raghubar KP, Barnes MA, Hecht SA. Working memory and mathematics: A review of developmental, individual difference, and cognitive approaches. Learn Individ Differ. 2010;20(2):110–22.

[7] Holmes J, Gathercole SE. Taking working memory training from the laboratory into schools. Educ Psychol. 2014 6 7;34(4):440–50. 10.1080/01443410.2013.797338 PMC4579053 26494933

[8] Holmes J, Gathercole SE, Dunning DL. Adaptive training leads to sustained enhancement of poor working memory in children. Dev Sci. 2009;12(4):F9–15. doi: 10.1111/j.1467-7687.2009.00848.x 19635074 10.1111/j.1467-7687.2009.00848.x

[9] Klingberg T, Forssberg H, Westerberg H. Training of Working Memory in Children With ADHD. J Clin Exp Neuropsychol Neuropsychol Dev. 2002;24(6):781–91. 10.1076/jcen.24.6.781.8395 12424652

[10] Melby-Lervåg M, Hulme C. Is working memory training effective? A meta-analytic review. Dev Psychol. 2013;49(2):270–91. doi: 10.1037/a0028228 22612437 10.1037/a0028228

[11] St Clair-Thompson H, Stevens R, Hunt A, Bolder E. Improving children’s working memory and classroom performance. Educ Psychol. 2010;30(2):203.

[12] Thorell LB, Lindqvist S, Bergman Nutley S, Bohlin G, Klingberg T. Training and transfer effects of executive functions in preschool children. Dev Sci. 2009;12(1):106–13. doi: 10.1111/j.1467-7687.2008.00745.x 19120418 10.1111/j.1467-7687.2008.00745.x

[13] Dunning DL, Holmes J, Gathercole SE. Does working memory training lead to generalized improvements in children with low working memory? A randomized controlled trial. Dev Sci. 2013;n/a–n/a. 10.1111/desc.12068 PMC4232921 24093880

[14] Redick TS, Shipstead Z, Wiemers EA, Melby-Lervåg M, Hulme C. What’s Working in Working Memory Training? An Educational Perspective. Educ Psychol Rev. 2015 12;27(4):617–33. doi: 10.1007/s10648-015-9314-6 26640352 10.1007/s10648-015-9314-6 PMC4667976

[15] Friso-van den Bos I, van der Ven SHG, Kroesbergen EH, van Luit JEH. Working memory and mathematics in primary school children: A meta-analysis. Educ Res Rev. 2013 12;10:29–44.

[16] Andersson U, Lyxell B. Working memory deficit in children with mathematical difficulties: A general or specific deficit? J Exp Child Psychol. 2007 3;96(3):197–228. doi: 10.1016/j.jecp.2006.10.001 17118398 10.1016/j.jecp.2006.10.001

[17] David CV. Working memory deficits in Math learning difficulties: a meta-analysis. Int J Dev Disabil. 2012;58(2):67–84.

[18] Swanson HL, Jerman O. Math disabilities: A selective meta-analysis of the literature. Rev Educ Res. 2006;76(2):249–74.

[19] De Rammelaere S, Stuyven E, Vandierendonck A. The contribution of working memory resources in the verification of simple mental arithmetic sums. Psychol Res. 1999;62(1):72–7.

[20] De Rammelaere S, Stuyven E, Vandierendonck A. Verifying simple arithmetic sums and products: Are the phonological loop and the central executive involved? Mem Cognit. 2001;29(2):267–73. 11352209 10.3758/bf03194920

[21] Furst AJ, Hitch GJ. Separate roles for executive and phonological components of working memory in mental arithmetic. Mem Cognit. 2000;28(5):774–82. 10983451 10.3758/bf03198412

[22] Hecht SA. Counting on working memory in simple arithmetic when counting is used for problem solving. Mem Cognit. 2002;30(3):447–55. 12061765 10.3758/bf03194945

[23] Hubber PJ, Gilmore C, Cragg L. The roles of the central executive and visuospatial storage in mental arithmetic: A comparison across strategies. Q J Exp Psychol. 2014;67(5):936–54. 10.1080/17470218.2013.838590 PMC4037843 24131334

[24] Imbo I, Vandierendonck A. Do multiplication and division strategies rely on executive and phonological working memory resources? Mem Cognit. 2007;35(7):1759–71. 18062552 10.3758/bf03193508

[25] Imbo I, Vandierendonck A. The role of phonological and executive working memory resources in simple arithmetic strategies. Eur J Cogn Psychol. 2007;19(6):910–33.

[26] Imbo I, Vandierendonck A. The development of strategy use in elementary school children: Working memory and individual differences. J Exp Child Psychol. 2007;96(4):284–309. doi: 10.1016/j.jecp.2006.09.001 17046017 10.1016/j.jecp.2006.09.001

[27] Lemaire P. The Role of working memory resources in simple cognitive arithmetic. Eur J Cogn Psychol. 1996;8(1):73.

[28] Baddeley AD. Exploring the central executive. Q J Exp Psychol. 1996;49A(1):5–28.

[29] Baddeley AD, Hitch GJ. Working memory In: Bower G A (ed), Recent advances in learning and motivation. New York: Academic Press; 1974.

[30] Daneman M, Carpenter PA. Individual differences in working memory and reading. J Verbal Learn Verbal Behav. 1980;19(4):450–66.

[31] Imbo I, Duverne S, Lemaire P. Working memory, strategy execution, and strategy selection in mental arithmetic. Q J Exp Psychol. 2007;60(9):1246–64. 10.1080/17470210600943419 17676556

[32] Duverne S, Lemaire P, Vandierendonck A. Do working-memory executive components mediate the effects of age on strategy selection or on strategy execution? Insights from arithmetic problem solving. Psychol Res. 2008 1 1;72(1):27 doi: 10.1007/s00426-006-0071-5 16838186 10.1007/s00426-006-0071-5

[33] Ashcraft MH. The development of mental arithmetic: A chronometric approach. Dev Rev. 1982 9;2(3):213–36.

[34] Bailey DH, Littlefield A, Geary DC. The codevelopment of skill at and preference for use of retrieval-based processes for solving addition problems: Individual and sex differences from first to sixth grades. J Exp Child Psychol. 2012 9;113(1):78–92. doi: 10.1016/j.jecp.2012.04.014 22704036 10.1016/j.jecp.2012.04.014 PMC3392429

[35] Imbo I, Vandierendonck A. Effects of problem size, operation, and working-memory span on simple-arithmetic strategies: differences between children and adults? Psychol Res. 2008;72(3):331–346. doi: 10.1007/s00426-007-0112-8 17457605 10.1007/s00426-007-0112-8

[36] Kaye DB, de Winstanley P, Chen Q, Bonnefil V. Development of Efficient Arithmetic Computation. J Educ Psychol. 1989;81(4):467–80.

[37] Hitch G, Cundick J, Haughey M, Pugh R, Wright H. Aspects of counting in children’s arithmetic. Cogn Process Math. 1987;26–41.

[38] McKenzie B, Bull R, Gray C. The effects of phonological and visual-spatial interference on children’s arithmetical performance. Educ Child Psychol. 2003;20(3):93–108.

[39] Thomas J, Zoelch C, Seitz-Stein K, Schumann-Hengsteler R. Phonological and central executive working memory processes in children’s mental addition and multiplication. Psychol Erzieh Unterr. 2006;53:275–90.

[40] Emerson MJ, Miyake A. The role of inner speech in task switching: A dual-task investigation. J Mem Lang. 2003;48(1):148.

[41] Pashler H. Dual-task interference in simple tasks: Data and theory. Psychol Bull. 1994;116(2):220–44. 7972591 10.1037/0033-2909.116.2.220

[42] Bayliss DM, Jarrold C, Gunn DM, Baddeley AD. The complexities of complex span: Explaining individual differences in working memory in children and adults. J Exp Psychol Gen. 2003;132(1):71–92. 12656298 10.1037/0096-3445.132.1.71

[43] Andersson U, Östergren R. Number magnitude processing and basic cognitive functions in children with mathematical learning disabilities. Learn Individ Differ. 2012 12;22(6):701–14.

[44] McLean JF, Hitch GJ. Working memory impairments in children with specific arithmetic learning difficulties. J Exp Child Psychol. 1999;74(3):240–60. doi: 10.1006/jecp.1999.2516 10527556 10.1006/jecp.1999.2516

[45] Schuchardt K, Maehler C, Hasselhorn M. Working Memory Deficits in Children With Specific Learning Disorders. J Learn Disabil. 2008 4 28;41(6):514–23. doi: 10.1177/0022219408317856 18625783 10.1177/0022219408317856

[46] Szűcs D, Devine A, Soltesz F, Nobes A, Gabriel F. Developmental dyscalculia is related to visuo-spatial memory and inhibition impairment. Cortex. 2013;49(10):2674–88. doi: 10.1016/j.cortex.2013.06.007 23890692 10.1016/j.cortex.2013.06.007 PMC3878850

[47] Szűcs D, Devine A, Soltesz F, Nobes A, Gabriel F. Cognitive components of a mathematical processing network in 9-year-old children. Dev Sci. 2014 7 1;17(4):506–24. doi: 10.1111/desc.12144 25089322 10.1111/desc.12144 PMC4253132

[48] Mix KS, Cheng Y-L. The Relation Between Space and Math: Developmental and Educational Implications. Benson JB, editor. Adv Child Dev Behav. 2012;42:197–243. 22675907 10.1016/b978-0-12-394388-0.00006-x

[49] Cragg L, Keeble S, Richardson S, Roome HE, Gilmore C. Direct and indirect influences of executive functions on mathematics achievement. Cognition. 2017;162:12–26. doi: 10.1016/j.cognition.2017.01.014 28189034 10.1016/j.cognition.2017.01.014

[50] Logie RH, Gilhooly KJ, Wynn V. Counting on working memory in arithmetic problem solving. Mem Cognit. 1994;22(4):395–410. 7934946 10.3758/bf03200866

[51] Dehaene S. Varieties of numerical abilities. Cognition. 1992;44(1–2):1–42. 1511583 10.1016/0010-0277(92)90049-n

[52] Chevalier N, Huber KL, Wiebe SA, Espy KA. Qualitative change in executive control during childhood and adulthood. Cognition. 2013 7;128(1):1–12. doi: 10.1016/j.cognition.2013.02.012 23562979 10.1016/j.cognition.2013.02.012 PMC4049232

[53] Kray J, Lindenberger U. Adult age differences in task switching. Psychol Aging. 2000;15:126–47. 10755295 10.1037//0882-7974.15.1.126

[54] Barrouillet P, Bernardin S, Camos V. Time constraints and resource sharing in adults’ working memory spans. J Exp Psychol Gen. 2004;133(1):83–100. doi: 10.1037/0096-3445.133.1.83 14979753 10.1037/0096-3445.133.1.83

[55] Barrouillet P, Lépine R. Working memory and children’s use of retrieval to solve addition problems. J Exp Child Psychol. 2005 7;91(3):183–204. doi: 10.1016/j.jecp.2005.03.002 15925643 10.1016/j.jecp.2005.03.002

[56] Wilson KM, Swanson HL. Are mathematics disabilities due to a domain-general or a domain-specific working memory deficit? J Learn Disabil. 2001;34(3):237–248. doi: 10.1177/002221940103400304 15499878 10.1177/002221940103400304

[57] Miyake A, Friedman NP, Rettinger DA, Shah P, Hegarty M. How are visuospatial working memory, executive functioning, and spatial abilities related? A latent-variable analysis. J Exp Psychol Gen. 2001 12;130(4):621 11757872 10.1037//0096-3445.130.4.621

[58] Gilmore C, Keeble S, Richardson S, & Cragg L. The interaction of procedural skill, conceptual understanding and executive functions in early mathematics achievement. Journal of Numerical Cognition. in press.

[59] Gilmore C, Attridge N, Clayton S, Cragg L, Johnson S, Marlow N, et al Individual differences in inhibitory control, not non-verbal number acuity, correlate with mathematics achievement. PLoS ONE. 2013 6 13;8(6):e67374 doi: 10.1371/journal.pone.0067374 23785521 10.1371/journal.pone.0067374 PMC3681957

