# Supplementary material for: When is working memory important for arithmetic? The impact of strategy and age
Source: PLoS One. 2017 Dec 11;12(12):e0188693. doi: 10.1371/journal.pone.0188693 (PMC5724815; doi:10.1371/journal.pone.0188693)
Supplement: S1 Appendix — (PDF) [file pone.0188693.s001.pdf]

Addition problems for the no load condition

Lists counterbalanced across strategies

1st addend	2nd addend	Sum	Cross decade boundary?
7	2	9	n
5	2	7	n
4	2	6	n
8	4	12	y
5	3	8	n
6	2	8	n
8	7	15	y
8	5	13	y
8	4	12	y
9	5	14	y
11	8	19	n
12	7	19	n
17	6	23	y
19	9	28	y
19	7	26	y
21	8	29	n
22	7	29	n
23	6	29	n
28	8	36	y
28	9	37	y

1st addend	2nd addend	Sum	Cross decade boundary?
6	2	8	n
7	2	9	n
8	3	11	y
4	3	7	n
9	5	14	y
5	3	8	n
6	3	9	n
7	4	11	y
8	2	10	n
9	7	16	y
12	6	18	n
14	5	19	n
16	5	21	y
18	7	25	y
24	4	28	n
25	4	29	n
25	9	34	y
27	6	33	y
28	7	35	y
28	6	34	y

1st addend	2nd addend	Sum	Cross decade boundary?
4	3	7	n
6	3	9	n
7	2	9	n
5	4	9	n
6	5	11	y
6	3	9	n
6	4	10	n
8	5	13	y
9	7	16	y
9	2	11	y
11	4	15	n
12	5	17	n
16	8	24	y
19	7	26	y
22	7	29	n
23	5	28	n
24	7	31	y
26	6	32	y
28	9	37	y
28	8	36	y

Addition problems for the control load condition

Lists counterbalanced across strategies

1st addend	2nd addend	Sum	Cross decade boundary?
6	2	8	n
5	2	7	n
5	3	8	n
5	4	9	n
6	3	9	n
7	5	12	y
9	7	16	y
8	7	15	y
8	2	10	n
9	3	12	y
13	8	21	y
14	3	17	n
19	4	23	y
21	5	26	n
23	6	29	n
24	3	27	n
25	6	31	y
25	8	33	y
27	4	31	y
28	7	35	y

1st addend	2nd addend	Sum	Cross decade boundary?
6	2	8	n
5	4	9	n
7	4	11	y
4	3	7	n
5	2	7	n
8	5	13	y
6	3	9	n
7	5	12	y
9	8	17	y
8	3	11	y
12	7	19	n
17	3	20	n
18	6	24	y
19	5	24	y
21	6	27	n
22	7	29	n
26	4	30	n
27	8	35	y
27	5	32	y
29	6	35	y

1st addend	2nd addend	Sum	Cross decade boundary?
6	2	8	n
4	2	6	n
9	5	14	y
5	4	9	n
6	4	10	n
6	2	8	n
8	7	15	y
7	5	12	y
9	8	17	y
9	4	13	y
13	7	20	n
15	3	18	n
16	3	19	n
18	5	23	y
19	7	26	y
21	7	28	n
23	7	30	n
28	6	34	y
28	7	35	y
29	5	34	y

Addition problems for the working memory load condition

Lists counterbalanced across strategies

1st addend	2nd addend	Sum	Cross decade boundary?
6	3	9	n
5	3	8	n
6	4	10	n
8	5	13	y
6	3	9	n
7	5	12	y
7	6	13	y
8	6	14	y
8	7	15	y
9	4	13	y
16	7	23	y
17	9	26	y
18	4	22	y
19	3	22	y
21	6	27	n
22	5	27	n
22	8	30	n
23	6	29	n
24	4	28	n
26	3	29	n

1st addend	2nd addend	Sum	Cross decade boundary?
6	3	9	n
5	4	9	n
4	3	7	n
8	5	13	y
6	3	9	n
6	7	13	y
9	7	16	y
7	2	9	n
8	3	11	y
9	4	13	y
13	7	20	n
14	5	19	n
16	4	20	n
17	9	26	y
19	7	26	y
23	6	29	n
24	6	30	n
24	7	31	y
26	8	34	y
27	8	35	y

1st addend	2nd addend	Sum	Cross decade boundary?
6	3	9	n
4	3	7	n
5	4	9	n
8	4	12	y
9	5	14	y
5	3	8	n
6	3	9	n
6	2	8	n
7	5	12	y
9	7	16	y
13	6	19	n
14	9	23	y
18	8	26	y
19	6	25	y
19	9	28	y
21	4	25	n
22	7	29	n
24	5	29	n
27	8	35	y
28	8	35	y
